# A New Method for Discovering Plant Biostimulants

**DOI:** 10.3390/plants13010056

**Published:** 2023-12-23

**Authors:** Peiwen Gao, Kui Wang, Chang Qi, Keming Chen, Wensheng Xiang, Yue Zhang, Jie Zhang, Changlong Shu

**Affiliations:** 1 State Key Laboratory for Biology of Plant Diseases and Insect Pests, Institute of Plant Protection, Chinese Academy of Agricultural Sciences, Beijing 100193, China; gaopeiwen@caas.cn (P.G.); qichang2053@126.com (C.Q.); cc256hh@163.com (K.C.); zhyue1028@163.com (Y.Z.); zhangjie05@caas.cn (J.Z.); 2 School of Plant Protection, Anhui Agricultural University, Hefei 230036, China; wangkui01@ahau.edu.cn; 3 College of Life Science, Northeast Agricultural University, HarBin 150030, China; xiangwensheng@neau.edu.cn; 4 Hebei Key Laboratory of Soil Entomology, Cangzhou Academy of Agricultural and Forestry Sciences, Cangzhou 061001, China

**Keywords:** biostimulant, humic acid, intestinal compound, piperic acid, *Protaetia brevitarsis*

## Abstract

Structurally well-defined compounds have advantages for quality control in plant biostimulant production and application processes. Humic acid (HA) is a biostimulant that significantly affects plant growth, and soil-dwelling *Protaetia brevitarsis* larva (PBLs) can rapidly convert agricultural waste into HA. In this study, we use PBLs as a model to investigate HA formation and screen for structurally well-defined HA-related plant biostimulant compounds. Dephasing magic angle spinning nuclear magnetic resonance (^13^C DD–MAS NMR) analysis indicated HA structural changes during PBL digestion; metabolic profiling detected seven HA-related aromatic ring-containing compounds. A total of six compounds that significantly stimulate plant growth were identified through plant experiments, and all six compounds demonstrate the ability to enhance seed germination. It is noteworthy that piperic acid exhibits a remarkable promotion of root growth in plants, a finding reported for the first time in this study. Thus, this study not only provides insights into the insect-mediated transformation of HA but also illustrates a new method for discovering structurally well-defined plant biostimulant compounds.

## 1. Introduction

The term plant biostimulants refers to substances or microorganisms that can stimulate plant nutrient processes independently of the nutrient content of the products [1]. They have the capacity to improve one or more characteristics of plants themselves or the root environment [2]. Currently, organic substances constitute a large category of plant biostimulants, and many organic compounds or organic extracts have been reported for having biological stimulating activity, such as humic acid (HA) [3,4,5]. However, these organic substances are mainly complex mixtures [6], which poses a challenge for quality control in subsequent production and application processes. Therefore, screening for structurally well-defined compounds that have plant stimulation activity is of great significance for the development, application, and mechanistic study of plant biostimulants.

HA is a type of plant biostimulant that is always applied to soil or plant roots [7,8]. According to the currently proposed soil humus formation and cycling models, such as “humification”, “selective preservation”, “progressive decomposition”, and “soil continuum model” [9], the precursors and decomposition products of HA coexist in soil and may also contribute to HA’s plant biostimulant activities. However, due to the low content of HA and the complexity of the substances in the soil [10,11], it is not easy to separate or accurately identify these precursors and decomposition products from soil.

It has been suggested that soil fauna plays an important role in litter decomposition and humification [12], and the larvae of the soil-dwelling insect *Protaetia brevitarsis* (PBLs) have been reported to feed on plant litter and form frass with a high HA content and whose ^13^C NMR spectrum is identical to that of soil HA [10]. Furthermore, PBLs can consume humus substances without soil [10,13,14,15]; thus, using this species can avoid noise in soil substances from impeding the identification of HA-related compounds, and PBLs may serve as a model to investigate HA formation and cycling. With the aim of screening compounds that can be used as plant biostimulants, in this investigation, we fed PBLs fermented corn straw, analysed the HA changes and identified metabolites from the digestive tract contents. Then, the aromatic compounds were selected for further assessment, and the plant biostimulant activity was assessed according to seed germination and growth-promoting effects. Not only will this research provide insights into the insect-mediated transformation of HA, but the discovery of structurally well-defined biostimulant compounds also offers valuable guidance for industrial-scale synthesis of plant biostimulant production.

## 2. Materials and Methods

### 2.1. Preparation of Fermented Corn Straw

The corn straw used in the experiment was collected from Fangshan, Beijing, and crushed to approximately 2 cm. Then, a compost pile with a square bottom of 4 m^2^ and a height of 70 cm was established to ferment the corn straw with 60% moisture content [16]. Approximately two months later, the temperature of the compost pile dropped, and the fermented corn stalks were air-dried for later use.

### 2.2. Insects and Feeding Conditions

The *Protaetia brevitarsis* (PB) population used in the experiment originated from a wild population in Gongzhuling, Jiling [17]. The 3rd instar larvae were used for the test, the body weight of the PBLs was between 1.8 g and 2.0 g, and the body length was approximately 3.0 cm. The PBLs were fed in a plastic box containing fermented corn stalks with a moisture content of 50–60% for feeding. They were kept in incubators with a relative humidity of 70%, a temperature of 25 °C, and a day/night ratio of 16 h:8 h.

### 2.3. Preparation of Samples

Healthy cultivated 3rd instar larvae were selected and immobilized on ice before dissection. After surface sterilization with sterile water—70% ethanol–sterile water sequentially, the midgut and hindgut of the larvae were dissected to obtain gut contents for further analysis [16]. To collect the frass, healthy cultivated PBLs were washed with sterile water and stored in an empty box at 25 °C for 2 days, and the frass was collected for further analysis [15].

### 2.4. Isolation and Structural Characterization of HA in Samples

The improved HA extraction method was used to separate HA components from samples. During the experiment, each sample was suspended in 0.1 M NaOH with a weight-to-volume ratio of 1:10 and oscillated at 220 rpm for 24 h at 37 °C. Centrifugation at 8000 rpm separated suspensions of different solubilities. The process was repeated twice, and the supernatant was mixed together. The extracted supernatant was acidified with 3 M HCl to pH 1.0 and stratified at room temperature for 24 h. After centrifugation at 8000 r/min, the precipitate obtained was the HA component [10]. The organic carbon structure of HA is represented by ^13^C NMR chemical shifts. It was characterized via ^13^C dipolar, dephasing magic angle spinning nuclear magnetic resonance (^13^C DD–MAS NMR) analysis. The analysis was carried out with a 600 MHz NMR spectrometer (JNM-ECZ600R, JEOL RESONANCE Inc., Tokyo, Japan) equipped with a 3.2 mm HXMAS probe at 12 kHz MAS. For each sample, the analysis ran for 11 h, and the relaxation lifetime was 10 s. The obtained ^13^C DD-MAS NMR spectra were then baseline-corrected and integrated into the following chemical shift regions: 0–44 ppm for alkyl carbon; 44–64 ppm for methoxycarbon; 64–93 ppm for alkoxy carbon; 93–142 ppm for aromatic carbon; 142–162 ppm for phenolic carbon; and 162–188 ppm for carboxyl carbon [10].

### 2.5. Identification of Organic Compounds in Digestive Tract Contents

Organic compound extraction: Approximately 30 mg of midgut or hindgut contents was suspended in 600 μL of methyl tertiary butyl ether (MTBE)/methanol/water (*v*/*v*/*v* = 5:16:4) solvent containing 3 μg/mL L-norvaline as an internal standard and homogenized in an ice bath by a TissueLyser (JX-24, Jingxin, Shanghai, China) with zirconia beads at 40 Hz for 3 min. Following centrifugation at 16,000× *g* and 4 °C for 15 min, a total of 480 μL of supernatant was collected. Another 360 μL methanol was added to the residue, the extraction process was repeated, and the supernatants from the two extractions were combined [18].

Derivatization: One hundred microlitres of combined supernatants and 10 μL of 50 μg/mL L-norleucine were mixed and evaporated to dryness under a nitrogen stream. The residue was reconstituted in 30 μL of 20 mg/mL methoxyamine hydrochloride in pyridine (containing 5 μg/mL of n-alkane standards), and the resulting mixture was incubated at 37 °C for 90 min. Then, 30 μL of BSTFA (with 1% TMCS) was added to the mixture and incubated at 70 °C for 60 min for further derivatization [18].

Gas chromatography–mass spectrometry (GC–MS) analysis: Instrumental analysis was performed using an Agilent 7890A/5975C GC–MS system (7890A/5975C, Agilent Technologies Inc., Santa Clara, CA, USA). An OPTIMA^®^ 5 MS Accent fused-silica capillary column (30 m × 0.25 mm × 0.25 μm; MACHEREY-NAGEL, Düren, Germany) was utilized to separate the derivatives [18].

### 2.6. Determination of the Seed Germination Index of Intestinal Compounds

Based on the possible compounds generated during lignin degradation and their related KEGG metabolic pathways, seven aromatic compounds were selected from the detection results. These compounds are vanillic acid, p-coumaric acid, piperic acid, catechol, hydroquinone, benzoic acid and p-hydroxybenzoic acid.

To evaluate the effect of these compounds on plant growth, we first determined the seed germination index (GI) using *Brassica campestris* L. Seed GI determination was conducted using a concentration of 40 μmol/L for the selected compounds based on their reported effective concentrations [19]. In 9-cm Petri dishes, three layers of sterilized filter paper were added, followed by the addition of 4 mL of the aforementioned culture solution. Each Petri dish contained 15 seeds of *B. campestris* L. For the control group, distilled water was used for treatment. Each treatment was replicated three times. After incubation in the dark at 26 °C for 4 days, the number of germinated seeds and the length of seedling roots were recorded. The formula for calculating the seed GI is shown below:

GI = (ANT × ALT)/(ANC × ALC) × 100%

where ANT represents the average number of germinated seeds in the experimental group, ALT represents the average root length of seeds in the experimental group, ANC represents the average number of germinated seeds in the control group, and ALC represents the average root length in the control group.

### 2.7. Screening the Concentration of Plant Growth-Promoting Intestinal Compounds

We then screened the concentration of plant growth-promoting intestinal compounds. *B. campestris* L. seeds were germinated in 15-cm Petri dishes. Two layers of sterilized filter paper were placed in the Petri dish, and 8 mL of distilled water was added to completely wet the filter paper. Healthy *B. campestris* L. seeds were evenly spread on the filter paper, and the dish was covered to maintain moisture. The Petri dishes were placed in a growth chamber for germination, with a temperature of 25 °C, humidity of 70% and a photoperiod of 16 h of light and 8 h of darkness, for approximately 48 h, until the hypocotyls had emerged from the seed coat by approximately 2 to 4 mm. *B. campestris* L. seedlings with consistent growth were selected for the next experiment.

The nutrient solution used in the experiment was Arabidopsis nutrient solution, and the total volume was 40 mL. The prepared culture medium was added to 200 g of river sand and mixed thoroughly. The concentration gradient of each compound in the culture medium was 0 μmol/L, 20 μmol/L, 40 μmol/L and 60 μmol/L, respectively. Healthy *B. campestris* L. seedlings were carefully selected and transplanted into the culture substrate with tweezers, with 3 replicates for each treatment and 5 seedlings per replicate. Then, the plants were cultured in a growth chamber with a temperature of 25 °C, humidity of 70%, and a photoperiod of 16 h of light and 8 h of darkness. After 36 h of cultivation, the *B. campestris* L. seedlings were carefully removed from the culture substrate and scanned to obtain images. Then, ImageJ was used to measure stem length and root length. The aboveground and belowground parts of the seedlings were separated, and their fresh weights were measured using a balance (CAV214C, Ohaus Instruments Ltd., Shanghai, China). The separated seedlings were then oven-dried at 80 °C, and the dry weights of the aboveground and root parts were measured.

### 2.8. Validation of the Plant Growth-Promoting Activity of Intestinal Compounds

To further verify the role of intestinal compounds in promoting plant growth, the peanut variety HY22 was used for pot experiments. Full peanut seeds, each 1.0 to 1.3 g, were selected and potted individually. Vermiculite was used as a cultivation substrate, 1/2 MS solution (pH natural) was used to supply nutrients, and 50 μmol/L intestinal compounds were used as stimulants. The pots were placed in the greenhouse and watered when needed, and the daily temperature was also recorded. After 15 days of culture, peanut seedlings were removed from the culture substrate and carefully cleaned. The relative leaf chlorophyll content was measured, and the aboveground dry weight and root dry weight were measured after oven-drying at 80 °C.

### 2.9. Statistical Analysis

Data analysis was performed using one-way ANOVA with a significance level of *p* < 0.05. Multiple comparisons were conducted using the LSD test when homogeneity of variance was met, and Dunnett’s T3 test was used for multiple comparisons when heterogeneity of variance was present.

### 2.10. Method Flow Chart

**Figure 1:**
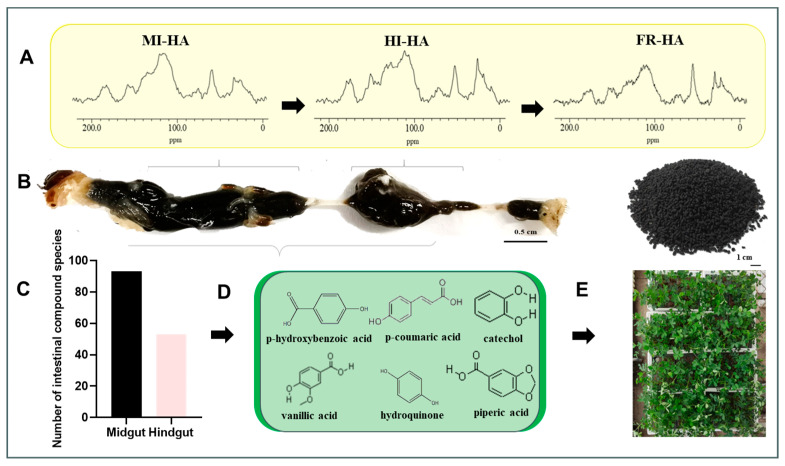
Method flow chart of discovering well-defined plant biostimulant compounds. The changes to HA structure in midgut inclusions (MI-HA), hindgut inclusions (HI-HA) and frass (FR-HA) (**A**). The view of the digestive tract of PBLs, showing relative locations of different compartments (**B**). Metabolomic analysis of midgut inclusions and hindgut inclusions showed that these compounds were distributed in both midgut and hindgut, with 93 compounds more distributed in the midgut and 53 compounds more distributed in hindgut (**C**). From the detected compounds, 7 compounds with benzene rings were selected for the seed germination experiment. Among them, 6 compounds could promote seed germination (**D**). The stimulating effects of 6 compounds on plant growth were further verified by plant growth experiments (**E**).

Method Flow Chart of Discovering Well-Defined Plant Biostimulant Compounds (Figure 1).

## 3. Results

### 3.1. Analysis of HA Structure in the Gut of PBLs

**Figure 2:**
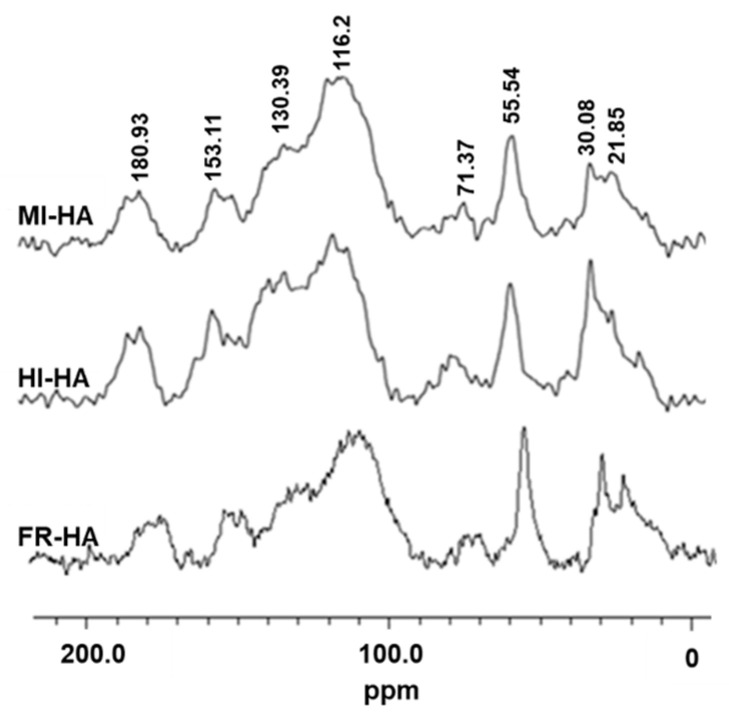
^13^C dipolar, dephasing magic angle spinning nuclear magnetic resonance spectra (^13^C DDMAS NMR spectra) of HA of midgut inclusions (MI-HA), hindgut inclusions (HI-HA) and the frass (FR-HA) of PBLs fed fermented corn stalks.

To analyse the structural changes of HA in the gut of PBLs feeding on fermented corn stalks, we used solid ^13^C DD-MAS NMR to characterize the HA from the gut inclusions and frass. From the obtained carbon spectrum (Figure 2), distinct structural differences between samples could be observed, and further integral analysis of the carbon spectrum showed the relative abundance changes of each functional group carbon. As shown in Table 1, during the transition from the midgut to the hindgut, the relative abundance of methyl carbon and aromatic carbon in HA decreased, while the relative abundance of alkyl carbon, phenolic carbon and carboxyl carbon increased; when transitioning from the hindgut to the frass, the relative abundance of alkyl carbon, methyl carbon, and aromatic carbon in HA increased, while alkoxy carbon, phenolic carbon and carboxyl carbon decreased. These results indicate that HA undergoes changes within the PBL gut and that HA precursors or decomposition products may coexist in the gut.

### 3.2. Organic Compounds in Gut Contents

**Figure 3:**
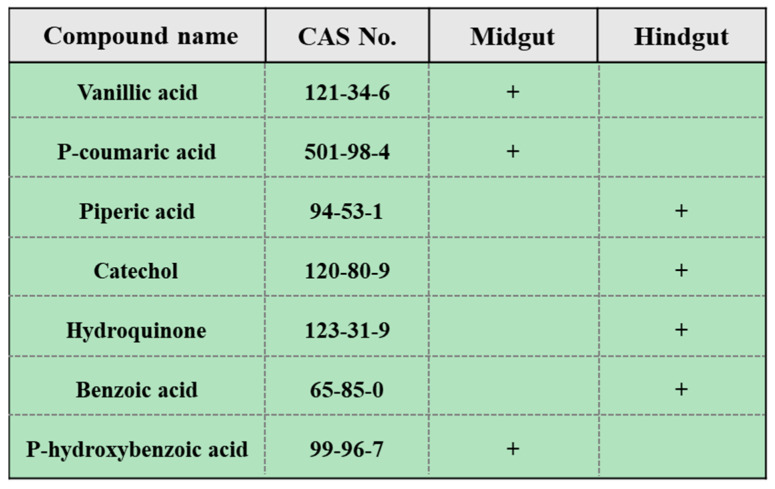
Seven benzene ring-containing compounds were selected in this study. “+” indicates a higher concentration in this gut.

To identify potential HA precursors in the gut of PBLs, an experimental detection and data analysis were conducted using a nontargeted metabolomics platform based on GC–MS. A total of 146 gut compounds were detected, primarily including alcohols, acids, sugars, esters and aromatic compounds, distributed in both the midgut and hindgut. Among them, 93 compounds were more prevalent in the midgut (Supplementary File S1), and 53 compounds were more prevalent in the hindgut (Supplementary File S1). Based on the possible types of compounds and relevant metabolic pathways in lignin degradation, seven benzene ring-containing compounds were selected from the detected compounds: catechol, p-coumaric acid, vanillic acid, piperic acid, benzoic acid, p-hydroxybenzoic acid and hydroquinone (Figure 3). The stimulating effects of vanillic acid, p-coumaric acid, catechol, hydroquinone and p-hydroxybenzoic acid on plant growth have been reported by researchers, but no promoting effects of piperic acid on plant growth have been reported.

### 3.3. Determination of the Seed GI of the Selected Compounds

**Figure 4:**
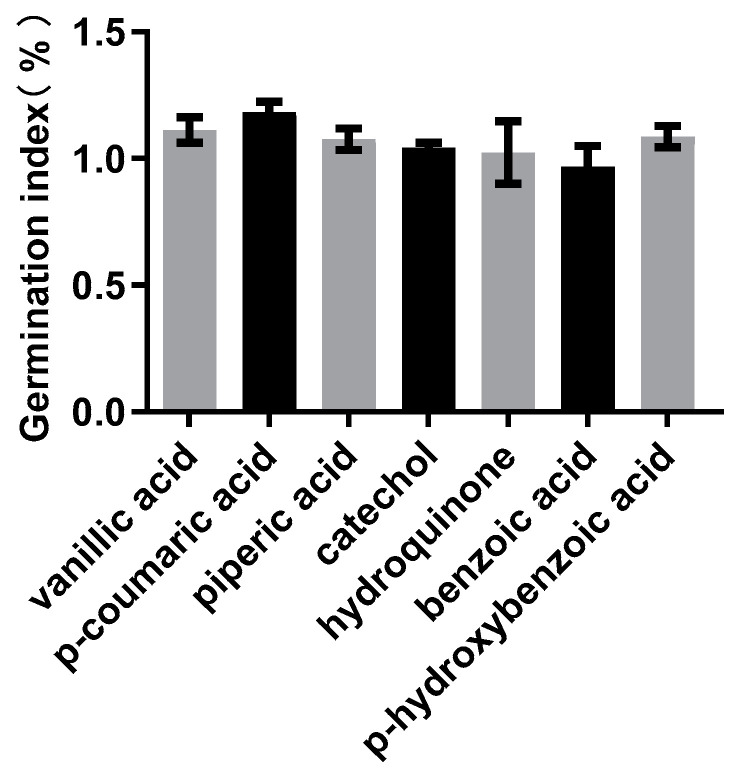
Seed germination index (GI) of seven compounds. GI > 1.0 indicates that the compound can promote seed germination.

To assess the effects of these compounds on plant germination, a GI test was initially conducted using rapeseed (canola). The results indicated that except for benzoic acid, the other six compounds showed the ability to promote seed germination at a concentration of 40 μmol/L (Figure 4). Thus, vanillic acid, p-coumaric acid, cinnamic acid, catechol, pyrocatechol and p-hydroxybenzoic acid were used for further testing.

### 3.4. Screening of the Plant Growth-Promoting Concentration of these Compounds

**Figure 5:**
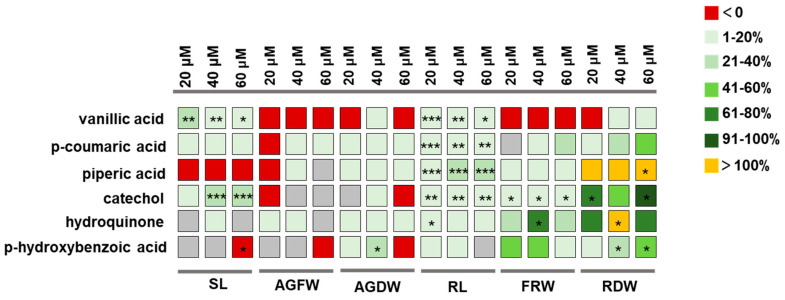
A summary of the promoting effects of six compounds on plants. SL indicates stem length; AGFW indicates aboveground fresh weight; AGDW indicates aboveground dry weight; RL indicates root length; FRW indicates fresh root weight; RDW indicates root dry weight. Each compound was administered at concentrations of 20, 40 and 60 μM. The different colours of the boxes represent the percentage increase in the assessed variable in the treatment group compared to the control group. *** *p* < 0.001, ** *p* < 0.01, * *p* < 0.05.

Further screening was conducted using sand culture experiments to determine the effective concentrations of the six gut compounds to promote plant growth. The results revealed that all six compounds showed growth-promoting effects on plants. Vanillic acid, catechol and p-hydroxybenzoic acid can promote growth in the aboveground part and root of the plant. On the other hand, piperic acid, hydroquinone and p-coumaric acid have specific growth-promoting effects on plant roots (Figure 5), and these growth-promoting effects are somewhat related to the concentration of the compounds. Vanillic acid showed a significant growth-promoting effect at 20~60 μmol/L, and the effect was more obvious at 20 μmol/L, and coumaric acid at the same concentration also showed a significant growth-promoting effect. In the concentration range of 20~60 μmol/L, piperic acid can significantly promote plant growth. In the range of 20 to 60 μmol/L, catechol had a significant effect on growth, and the effect was more obvious at 40 μmol/L and 60 μmol/L. Hydroquinone can significantly promote plant growth in the concentration range of 20–40 μmol/L. P-hydroxybenzoic acid also has a significant growth-promoting effect at 40–60 μmol/L, for which the effect is more pronounced at 40 μmol/L (Supplementary File S2: Figures S1–S6).

### 3.5. Validation of the Plant Growth-Promoting Activity

**Figure 6:**
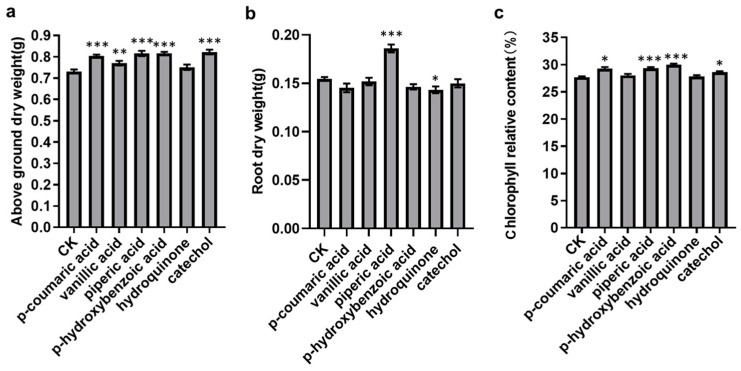
Functional verification results of 6 compounds promoting plant growth. (**a**–**c**) represent the effects of 6 compounds on aboveground dry weight, root dry weight and the relative chlorophyll content, respectively. *** *p* < 0.001, ** *p* < 0.01, * *p* < 0.05. CK means the control treatment.

To validate the plant growth-promoting effects of these compounds, based on the data results from Section 3.4, we selected a compound concentration of 50 μmol/L and conducted a pot experiment using peanut. Growth-related traits including aboveground dry weight, root dry weight and chlorophyll relative content were measured. Statistical analysis of variance and multiple comparisons were employed to confirm the growth-promoting capabilities of these compounds. As shows in Figure 6, the results indicated that all six selected compounds have the ability to increase the aboveground dry weight of peanut seedlings and that p-coumaric acid, vanillic acid, piperic acid, p-hydroxybenzoic acid and catechol exhibit a significant promoting effect. However, the root dry weight data shows that only piperic acid has a significant promoting effect, while the other five compounds exhibit an inhibitory effect.

Furthermore, the study also found that all six compounds can elevate the relative chlorophyll content of peanut seedlings. Among them, p-coumaric acid, piperic acid, p-hydroxybenzoic acid and catechol significantly increase the relative chlorophyll content. We observed a positive correlation between the increase in chlorophyll content and the increase in aboveground dry weight, possibly because the higher chlorophyll content is conducive to photosynthesis, thereby increasing the aboveground dry weight.

## 4. Discussion

Soil-dwelling insects play a crucial role in the carbon cycle of terrestrial ecosystems because they assist in the breakdown and decomposition of cellulose and lignocellulosic components within biomass [12]. Research has shown that in natural environments, soil-dwelling insects can rapidly degrade and convert plant residues with high lignin content into humic substances within a matter of hours. In contrast, microorganisms typically require several months or even years to transform plant residues into humic substances. Soil-dwelling insects can degrade efficiently by participating in a series of processes, including fragmentation, microbial consumption, inducing changes in pH and redox conditions, removing easily degradable polysaccharides, increasing lignin proportions, and reducing levels of soluble polyphenols and carbon-to-nitrogen ratios. Through these biotic disruption processes, soil-dwelling insects can convert plant residues into nutrient-rich mull-type HA, while humic substances produced solely through microbial action are referred to as more-type HA [12,20,21]. Consequently, soil-dwelling insects play a pivotal role in the transformation of plant residues into mull-type HA. In China, soil-dwelling insects, such as PBLs, are used as a resource and have been successfully employed to convert plant residues into humic acid-rich frass products [10,22]. Based on these data, the innovative strategy in this study uses PBLs that feeds on fermented maize straw as a model to screen compounds to use as plant biostimulants.

PBLs are an easily reared soil-dwelling insects that feed on decaying plant litter. Their digestive system is divided into three main parts: the foregut, located at the front end of the digestive tract, primarily serves as a food storage compartment; the midgut occupies a significant portion of the larva’s body cavity and has an elongated tubular structure. The midgut fluid exhibits strong alkaline properties with a high pH value of approximately 10 to 11, creating a favourable environment for the dissolution and decomposition of organic matter. The hindgut is cylindrical in shape and maintains a neutral pH of around eight. Within the hindgut is a rich and active microbial community that plays a crucial role in the further fermentation of organic materials [10,14,15,17]. Recent studies have found that due to their unique physicochemical properties in the gut and synergistic interactions with gut microbiota, PBLs can effectively degrade corn straw and produce frass-containing HA substances [10,16]. Previous research has suggested the crucial role of lignin and its degradation products in the formation of HA [23,24], while lignin is an aromatic polymer that contains a large number of benzene rings [25]. In this study, we have discovered that the digestive contents of PBLs show a high concentration of compounds containing benzene rings after a diet of fermented corn stalks (Supplementary Material S1), and the HA structure changes as it moves through the PBL intestinal tract (Figure 2, Table 1). These data indicated that, under the action of microbiota, HA in the PBL gut is continuously forming and decomposing, similar to the process described in the “soil continuum model” [9]. As such, screening compounds related to HA derived from PBL gut contents is a logical strategy for developing biostimulants.

Currently, many compounds with benzene rings have been reported to have plant biostimulation activity. Previous reports have shown that catechol can induce primary root growth by altering gene expression in the reactive oxygen species (ROS) pathway and regulating the distribution of H_2_O_2_ in the root elongation zone of plants [26]. Furthermore, catechol can also enhance a plant’s antifungal capabilities and boost its disease resistance by increasing the activity of peroxidases and polyphenol oxidases within the plant [27]. Exogenous p-coumaric acid has the ability to promote plant growth and enhance its growth performance by activating the active oxygen signalling pathway involving O^2·−^ [28]. Additionally, exogenous vanillic acid can strengthen a plant’s salt tolerance and growth performance by participating in the plant’s antioxidant defence mechanisms and the aldehyde dehydrogenase system [19]. Low concentrations of hydroquinone can stimulate seed germination and the growth of seedlings while concurrently inhibiting the growth of fungal pathogens on seeds. The mechanism of action may be associated with redox reactions between quinones and phenols [29,30,31]. Similarly, p-hydroxybenzoic acid can promote seed germination and the growth of seedlings at low concentrations, and it can also affect the activity of the plant’s antioxidant enzymes [32,33]. In this study, 7 compounds with benzene rings were selected based on the metabolome analysis of PBL midgut and hindgut inclusions. The plant determination results showed that vanillic acid, p-coumaric acid, cinnamic acid, catechol, pyrocatechol and p-hydroxybenzoic acid had good germination promotion and significant growth promotion activity. The promoting effect of these compounds on plants may be the reason why HA can serve as a biostimulant. Furthermore, it is worth noting that this study first reported the growth-stimulating effect of piperic acid on plants, even though it is known to play important roles as an antioxidant, antimicrobial and anti-inflammatory agent in the food and pharmaceutical fields [34,35,36]. In this report, we assume that it also acts as a plant antioxidant; however, the details of the specific mechanisms of action still require further comprehensive research.

## 5. Conclusions

In summary, this study illustrates a new method to discover well-defined plant biostimulant compounds (Figure 1). It not only provides insights into insect-mediated transformation of HA but also identified six compounds with well-defined structures that have growth-stimulating effects and reported for the first time the biostimulatory activity of piperic acid. Therefore, this work will contribute to advancing industrial synthetic biostimulant production.

## Figures and Tables

**Table 1:** The relative abundances of different carbon groups in HA.

	% Alkyl C (0–44 ppm)	% Methoxy C (44–64 ppm)	% Alkoxy C (64–93 ppm)	% Aromatic C (93–142 ppm)	% Carboloy C (142–162 ppm)	% Carboxyl C (162–188 ppm)
MI-HA	15.08	10.09	5.21	57.62	5.97	6.04
HI-HA	17.77	8.36	5.11	50.21	11.03	7.52
FR-HA	19.42	10.40	2.96	53.03	7.44	6.77

MI-HA: HA of midgut inclusions; HI-HA: HA of hindgut inclusions; FR-HA: HA of the frass.

## Data Availability

Data are contained within the article and supplementary materials.

## Supplementary Materials

The following supporting information can be downloaded at: https://www.mdpi.com/article/10.3390/plants13010056/s1.
