# Supplementary material for: A New Method for Discovering Plant Biostimulants
Source: Plants (Basel). 2023 Dec 23;13(1):56. doi: 10.3390/plants13010056 (PMC10780382; doi:10.3390/plants13010056)
Supplement: Supplementary file 1 [file plants-13-00056-s001.zip › Supplementary File S2.pdf]

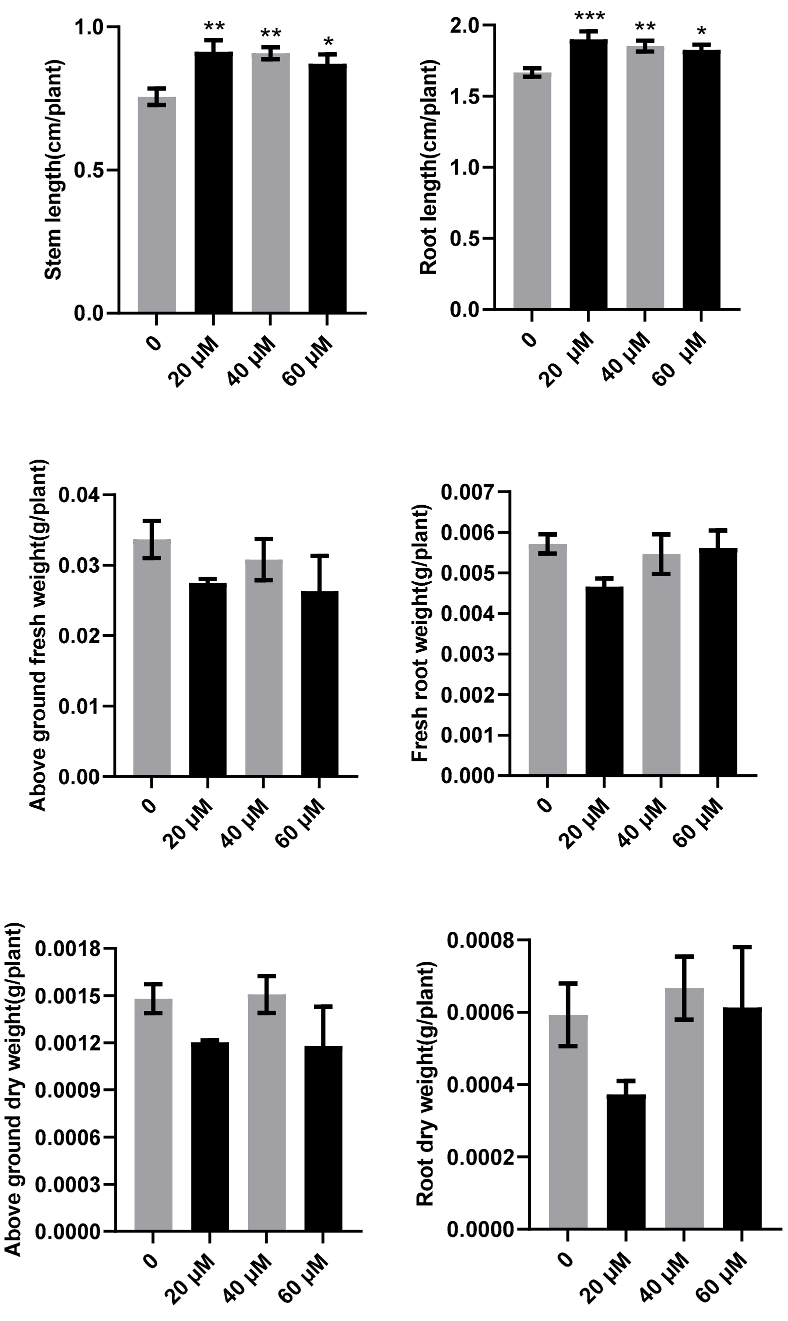

Figure S1. Concentration screening of vanillic acid for promoting plant growth. When the concentration of vanillic acid was 20 μmol/L, the root length of seedlings significantly increased, indicating that vanillic acid had a promoting effect on plant growth at 20 μmol/L. *** $P < 0.001$, ** $P < 0.01$, * $P < 0.05$.

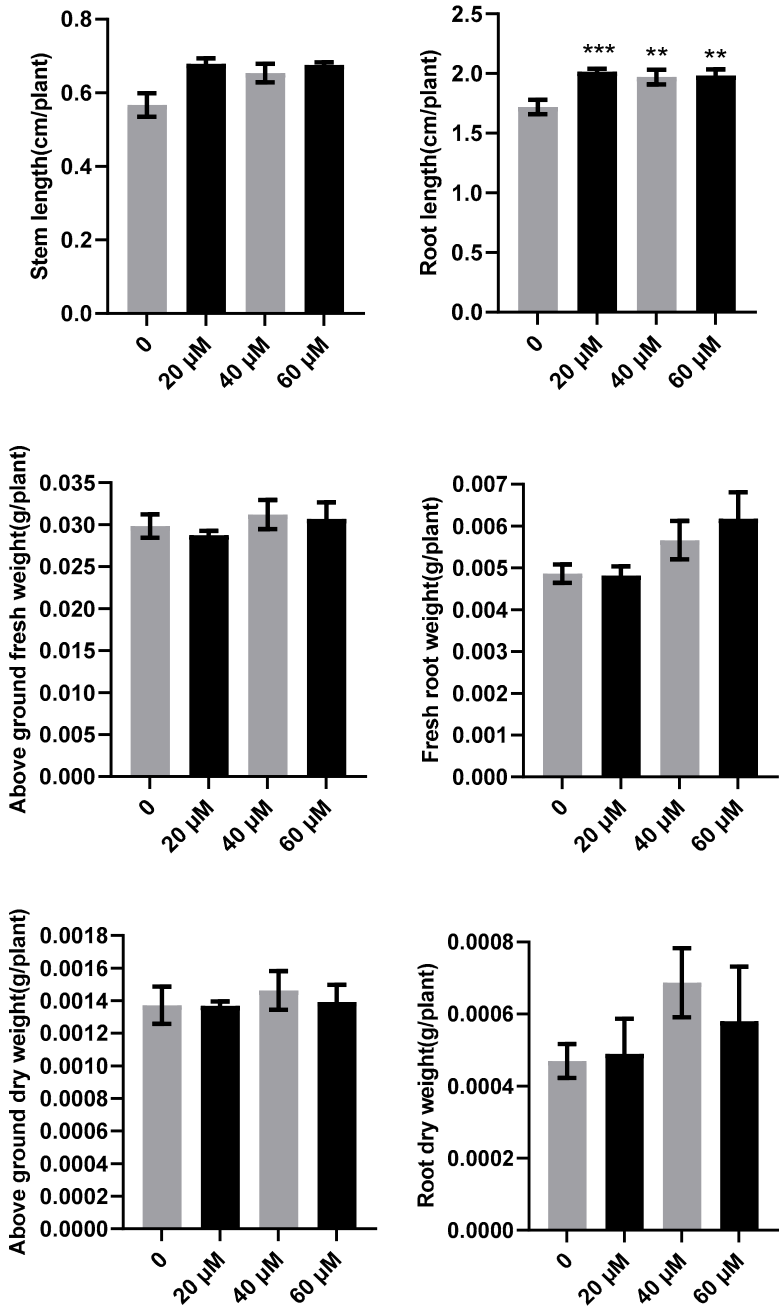

Figure S2. Concentration screening of p-coumaric acid for promoting plant growth. The root length of seedlings significantly increased when the concentration of p-coumaric acid was 20 μmol/L, indicating that p-coumaric acid had a promoting effect on plant growth at 20 μmol/L. ** $P < 0.01$.

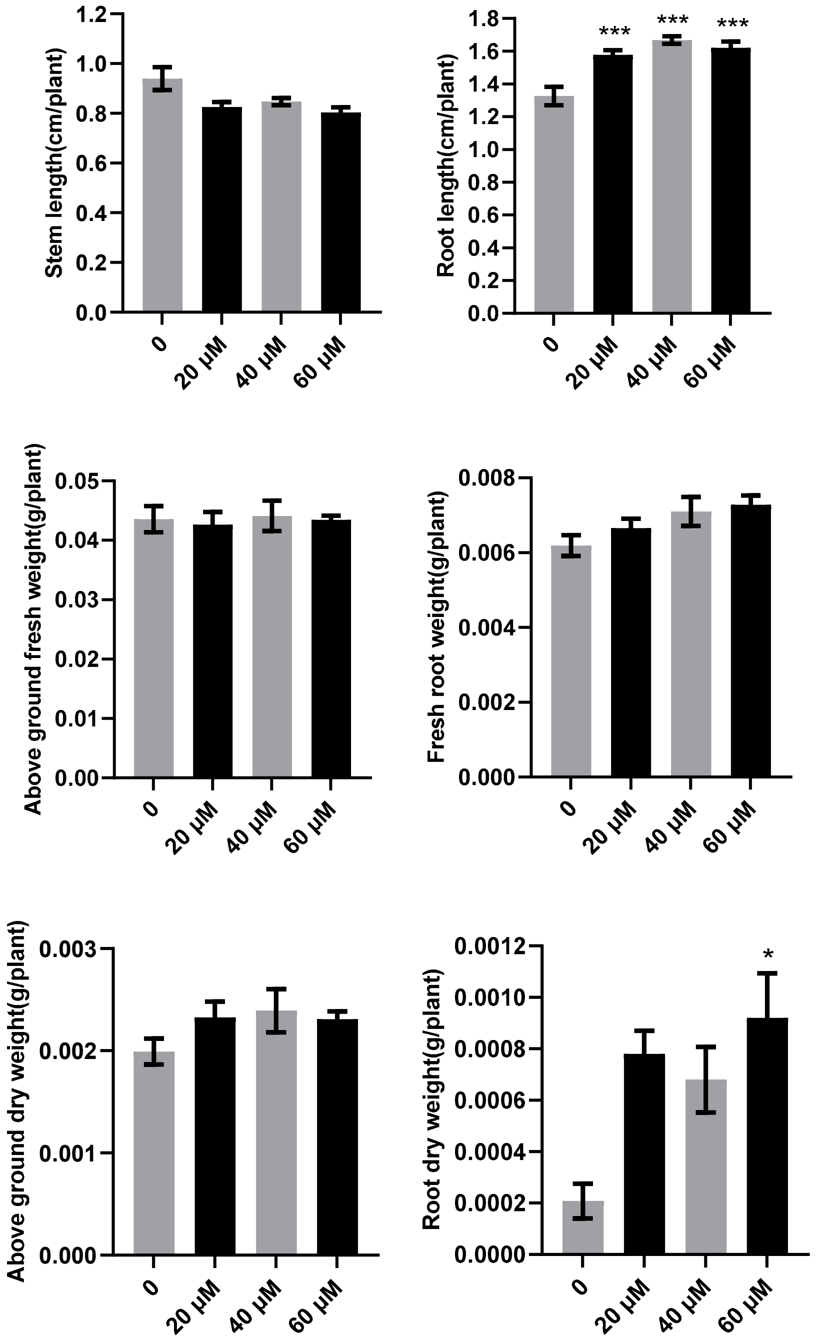

Figure S3. Concentration screening of piperic acid for promoting plant growth. Piperic acid at 20 μmol/L-60 μmol/L significantly increased the root length of seedlings, and 60 μmol/L significantly increased the root dry weight of seedlings, indicating that piperic acid at 20 μmol/L-60 μmol/L significantly promoted plant growth. The effect was more obvious at 60 μmol/L. *** $P < 0.001$, * $P < 0.05$.

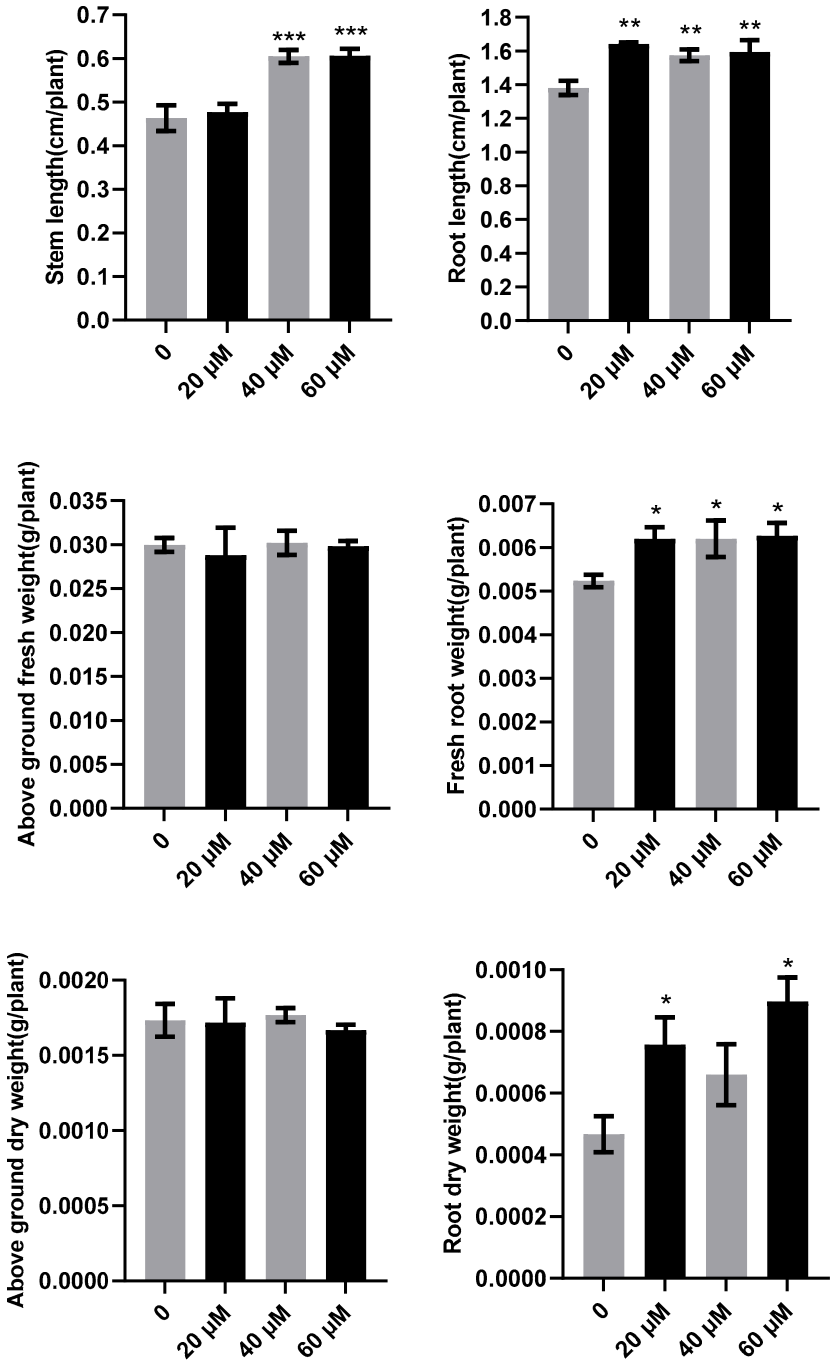

Figure S4. Concentration screening of catechol for promoting plant growth. The root length, fresh root weight and dry root weight of the seedlings at 20 μmol/L and 60 μmol/L were significantly higher than those of the control group. The root length significantly increased at 40 μmol/L, and the stem length significantly increased at 60 μmol/L. These results indicate that catechol can significantly promote plant growth when the concentration is 20 μmol/L-60 μmol/L. *** $P < 0.001$, ** $P < 0.01$, * $P < 0.05$.

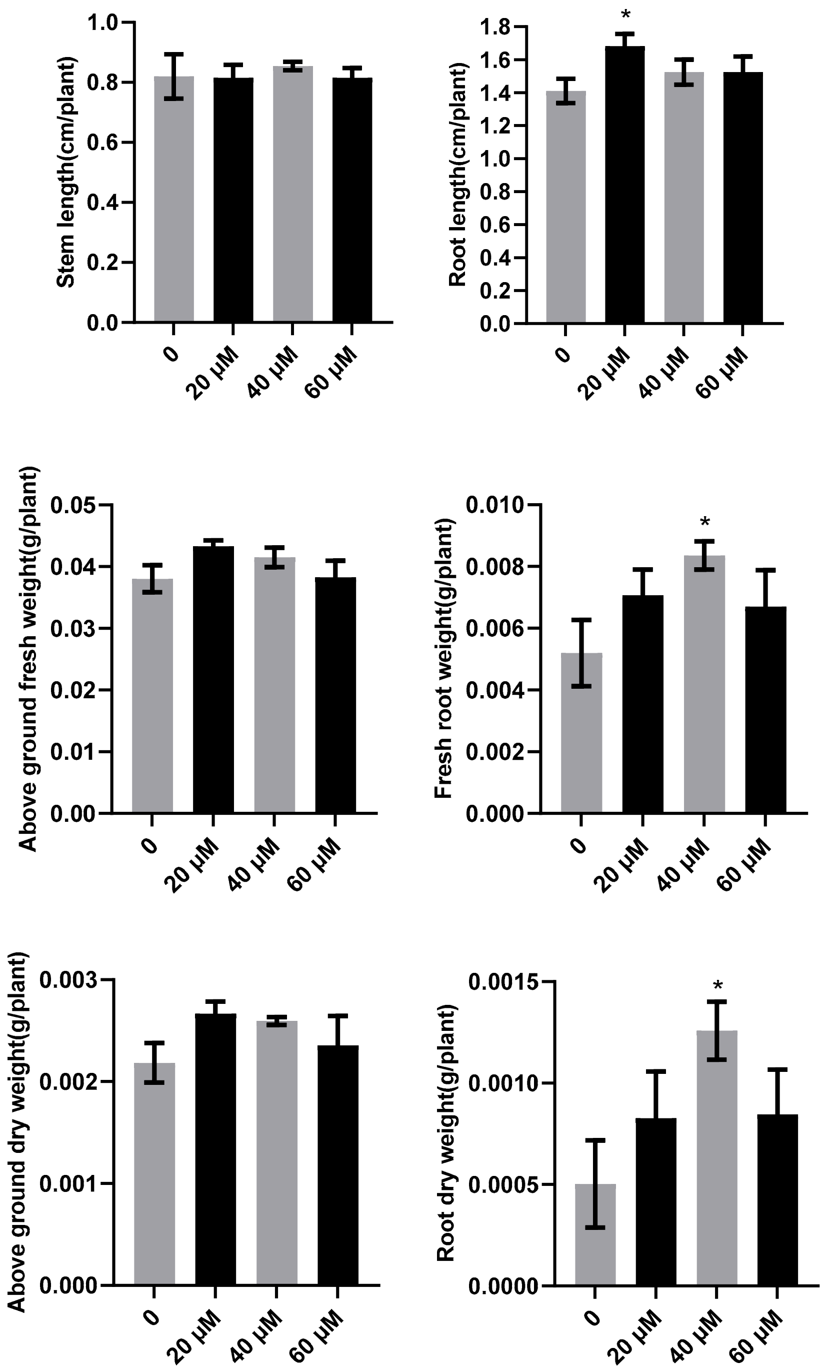

Figure S5. Concentration screening of hydroquinone for promoting plant growth. Hydroquinone at 20 μmol/L significantly increased the root length of seedlings, and 40 μmol/L significantly increased the fresh root weight and dry root weight of seedlings, indicating that hydroquinone at 20 μmol/L and 40 μmol/L significantly promoted the growth of plants. * $P < 0.05$.

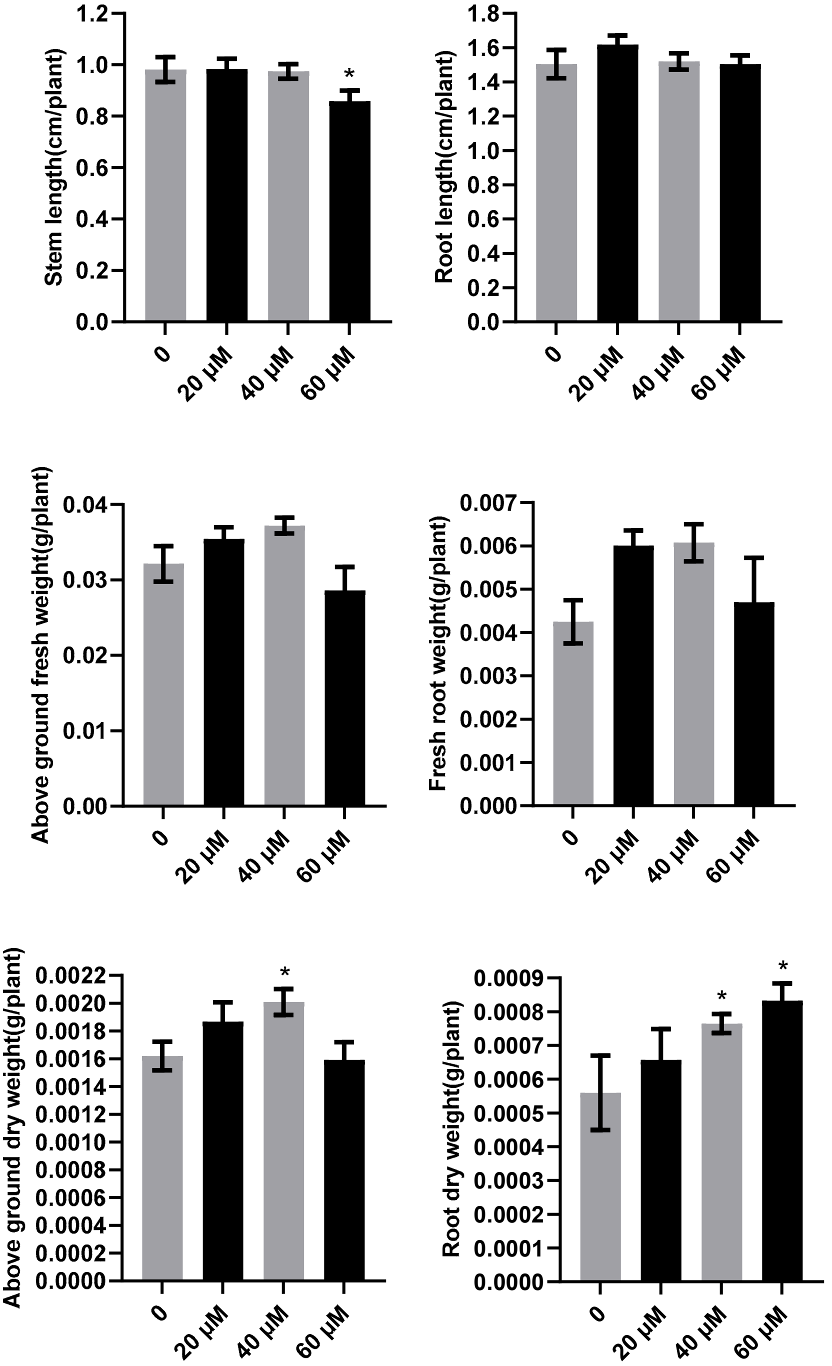

Figure S6. Concentration screening of p-hydroxybenzoic acid for promoting plant growth. P-hydroxybenzoic acid significantly increased the stem length of seedlings at 20 μmol/L and 40 μmol/L, significantly increased the root dry weight of seedlings at 40 μmol/L and 60 μmol/L, and significantly increased the aboveground dry weight of seedlings at 40 μmol/L but decreased the stem length at 60 μmol/L. These results indicate that p-hydroxybenzoic acid can significantly promote plant growth at 20 μmol/L and 40 μmol/L, and the effect is most obvious at 40 μmol/L. * $P < 0.05$.
